# Identification of a novel five ferroptosis-related gene signature as a promising prognostic model for breast cancer

**DOI:** 10.1007/s00432-023-05423-5

**Published:** 2023-09-20

**Authors:** Tian- cheng Cheng, Jia-hao Wu, Bei Zhu, Hai-yan Gao, Lin Zheng, Wei-xian Chen

**Affiliations:** 1 Department of Breast Surgery, The Affiliated Changzhou No. 2 People’s Hospital of Nanjing Medical University, 29 Xinglongxiang, Changzhou, 213000 Jiangsu Province China; 2 Graduate School, Bengbu Medical College, Bengbu, 233000 Anhui Province China; 3 Graduate School, Dalian Medical University, Dalian, 116000 Liaoning Province China; 4 Department of Breast Surgery, The Affiliated Changzhou Tumor Hospital of Soochow University, Changzhou, 213000 Jiangsu Province China; 5 Post-Doctoral Working Station, The Affiliated Changzhou No. 2 People’s Hospital of Nanjing Medical University, ChangzhouJiangsu Province, 213000 China

**Keywords:** Breast cancer, Ferroptosis, Gene signature, Overall survival, Prognosis

## Abstract

**Background:**

Breast cancer (BCa) is a major challenge for women’s health worldwide. Ferroptosis is closely related to tumorigenesis and cancer progression. However, the prognostic value of ferroptosis-related genes in BCa remains unclear, and more accurate prognostic models are urgently needed.

**Methods:**

Gene expression profiles and clinical information of BCa patients were collected from public databases. LASSO and multivariate Cox regression analysis were utilized to construct the prognostic gene signature. Kaplan–Meier plotter, receiver operating characteristic (ROC) curves, and nomogram were used to validate the prognostic value of the gene signature. Gene set enrichment analysis was performed to explore the molecular functions and signaling pathways.

**Results:**

Differentially expressed ferroptosis-related genes between BCa samples and normal tissues were obtained. A novel five-gene signature including BCL2, SLC40A1, TFF1, APOOL, and PRAME was established for prognosis prediction. Patients stratified into high-risk or low-risk group displayed significantly different survival. Kaplan–Meier and ROC curves showed a good performance for survival prediction in different cohorts. Biological function analysis revealed that the five-gene signature was associated with cancer progression, immune infiltration, immune response, and drug resistance. Nomogram including the five-gene signature was established.

**Conclusion:**

A novel five ferroptosis-related gene signature and nomogram could be used for prognostic prediction in BCa.

**Supplementary Information:**

The online version contains supplementary material available at 10.1007/s00432-023-05423-5.

## Introduction

Breast cancer (BCa) is the most frequently diagnosed malignancy and a leading cause of cancer-related death among females worldwide (Giaquinto et al. 2022). Systematic treatments including surgical resection, adjuvant chemotherapy, accurate radiotherapy, endocrine agents, biological targeting drugs, and even immune regulators have greatly improved the overall survival, but prognosis of BCa patients remains poor. While clinical, pathological, and molecular features are widely used in predicting treatment response and clinical outcomes, finding more sensitive and effective biomarkers as surrogates of these features and building more accurate prognostic models are of crucial importance in BCa research (Duffy et al. 2015).

Ferroptosis is a new form of programmed cell death caused by the accumulation of lipid-based reactive oxygen species, and is morphologically, biochemically, and genetically different from apoptosis, necrosis, and autophagy (Dixon et al. 2012; Stockwell et al. 2017). Emerging evidence indicated that ferroptosis is closely related to tumorigenesis and cancer progression, induction of ferroptosis process has become a novel therapeutic strategy in cancer treatment (Xu et al. 2019). Although precise physiological functions of ferroptosis have yet to be extensively investigated, role and molecular mechanisms of ferroptosis in BCa have been investigated in several studies. For example, Yang et al. demonstrated the ferroptosis landscape of triple-negative BCa and revealed an innovative immunotherapy combination strategy for clinical treatment (Yang et al. 2023). Yu et al. found that BCa stem cells could protect metastasized cancer cells from ferroptosis by regulating phenotypic plasticity (Wu et al. 2022). Several ferroptosis-related genes such as ACSL4, GPX4, P53, and SLC7A11 have been reported to be promising targets in BCa treatment, but the expression patterns and prognostic values of ferroptosis-related genes in BC remain unclear (Li et al. 2020). Studies with regard to ferroptosis-related gene signature as a prediction model are emerging. Liang et al. identified a ten ferroptosis-related gene signature to predict overall survival for hepatocellular carcinoma (Liang et al. 2020). A 19 ferroptosis-related gene signature established by Liu et al. was able to predict glioma cell death and glioma patient progression (Liu et al. 2020).

In this study, gene expression profiles and clinical information of BCa patients were collected from public databases. Novel ferroptosis-related genes were identified, a prognostic five-gene signature and nomogram were established to predict overall survival of BCa. Gene set enrichment analysis was performed to explore the molecular functions and signaling pathways. The findings of the present work might offer useful insights in finding effective biomarkers for BCa prognosis prediction and building accurate prognostic model in clinical practice.

## Materials and methods

### Data collection

The mRNA sequencing data, survival time, and corresponding clinical and molecular information of BCa patients were obtained from the TCGA database (https://portal.gdc.cancer.gov/). These level 3 data are publicly available for anyone to download and analyze during cancer research. Patients with no prognostic information or incomplete gene expression data were excluded to ensure accurate measurement. GSE20685, GSE20711, GSE42568, and GSE131769 datasets were downloaded from the GEO database (https://www.ncbi.nlm.nih.gov/gds/) for validation. Moreover, a total of 520 ferroptosis-related genes were retrieved from previous publications and are listed in Supplementary Table S1.

### Clustering of ferroptosis-related genes

R, a language and environment for statistical computing and graphics (version 3.6.3, http://www.R-project.org/), offers a wide variety of statistical and graphical techniques. In the present work, consensus clustering was performed via PAM method (K-medoids) using the ConsensusClusterPlus package in R language. The optimal cluster “K” was determined using the cumulative distribution function (CDF), and number of groups was identified based on relative CDF delta area plot stability as previously described (Cancer Genome Atlas Research Network 2017).

### Identification of ferroptosis-related genes

Differentially expressed genes between selected ferroptosis clusters were identified using the limma packages in R language. They were defined according to the criterion: |log_2_FC|> log_2_(1.5), false discovery rate < 0.05.

### Construction of a prognostic model based on ferroptosis-related genes

Prognostic model was constructed according to the preliminarily screened gene dataset. Differentially expressed ferroptosis-related genes were further narrowed down via the least absolute shrinkage and selection operator (LASSO) analysis using the glmnet package in R language. Risk score for each patient was calculated based on a linear combination of expression values (weighted by the coefficient of a multivariate Cox regression analysis).

$${\text{Risk}} {\text{Score}} = \mathop \sum \limits_{i = 1}^{n} {\text{Coefficient }}i \times {\text{Expression}} i$$

Coefficient *i* represents the coefficient of relative prognostic ferroptosis-related genes in multivariate Cox regression model, and expression *i* stands for the expression level of each ferroptosis-related genes. Patients were stratified into high-risk or low-risk group based on the cutoff value of risk score.

### Validation of a prognostic model based on ferroptosis-related genes

To validate such prognostic model, patients in the TCGA, GSE20685, GSE20711, GSE42568, and GSE131769 cohorts were also divided into high-risk or low-risk group using the same method as described above, followed by Kaplan–Meier analysis and receiver operating characteristic (ROC) curve analysis.

### Analysis of the biological functions associated with gene signature

Selected genes were uploaded to the Search Tool for the Retrieval of Interacting Genes (STRING) database (version: 11.5, http://www.string-db.org/) and GeneMANIA database (version: 2.6, https://genemania.org/) to assess protein–protein interaction information. Gene Ontology (GO) and Kyoto Encyclopedia of Genes and Genomes (KEGG) pathway enrichment analysis were performed using the clusterProfiler package in R software. Survival curves were plotted using the online Kaplan–Meier plotter (last updated August 10, 2023, http://kmplot.com/analysis/). Immune infiltration, methylation, drug sensitivity, copy number variation, miRNA regulatory network, and pathway activity were analyzed using the GSCA database (last updated April 4, 2023, http://bioinfo.life.hust.edu.cn/GSCA/) and GSCALite database (last updated April 4, 2023, http://bioinfo.life.hust.edu.cn/web/GSCALite/).

### PCR validation and HPA database

A total of five pairs of BCa samples and normal breast tissues were obtained from the Affiliated Changzhou No. 2 People’s Hospital of Nanjing Medical University. Collection, preservation, and analyzation of clinical samples were conducted in accordance with the Declaration of Helsinki and approved by the ethics committee of Changzhou No. 2 People’s Hospital. Informed written consent was received from all patients. In brief, TRIzol reagent (Thermo Fisher Scientific, USA) was used to extract total RNA, and Hiscript II qRT SuperMix (Vazyme, China) was applied to transcribe RNA into cDNA. Quantitative real‐time PCR was then performed using the AceQ qPCR SYBR Green Master Mix (Vazyme, China) on a ViiA 7 Real-Time PCR System (Thermo Fisher Scientific, USA). Primer sequences used for PCR are shown in Table 1. All reactions, including the negative controls, were performed in triplicate.

**Table 1:** Primer sequences used for PCR

Gene	Primer	Sequence (5' → 3')
BCL2	Forward primer	ATCGCCCTGTGGATGACTGA
	Reverse primer	ACAGCCAGGAGAAATCAAACAGA
APOOL	Forward primer	TTACAGTTTCAGGATTGGCG
	Reverse primer	ATACCTTTTTTGCTGTTACCTTAG
SLC40A1	Forward primer	GTCATCGGCTGTGGCTTTA
	Reverse primer	TTTCAATTCAGTTTCCTCTTCTTTA
PRAME	Forward primer	GGGTTCCATTCAGAGCCGATA
	Reverse primer	GCTGTGTCTCCCGTCAAAGG
TFF1	Forward primer	CCCCCGTGAAAGACAGAA
	Reverse primer	CGATGGTATTAGGATAGAAGCAC
β-actin	Forward primer	TCAAGATCATTGCTCCTCCTGAG
	Reverse primer	ACATCTGCTGGAAGGTGGACA

With respect to histological level, Human Protein Atlas (HPA) database (version: 23.0, https://www.proteinatlas.org/) is a proteomic tool for visualizing the distribution of protein expressions across most common cancers. In the present study, HPA was used to compare the protein expression levels of ferroptosis-related prognostic genes.

### Building and validating the predictive nomogram

Nomogram is widely used to predict cancer prognosis. All independent prognostic factors identified by univariate and multivariate Cox regression analysis were selected to establish a nomogram. ROC curves and calibration plot of the long-term survival probability examined the accuracy of nomogram predictions. In the present work, nomogram was generated to predict 3-year, 5-year, and 7-year overall survival of BCa using the RMS package in R language.

### Statistical analysis

All statistical analyses were performed using R software (version 3.6.3) with several packages including limma (version 3.40.6), clusterProfiler (version 3.14.3), maxstat (version 0.7–25), and pROC (version 1.17.0.1). Student's *t* test or Chi-square test was used to determine differences between variables. Statistical significance was assumed when *P* < 0.05.

## Results

### Clustering and identification of ferroptosis-related genes

**Fig. 1:**
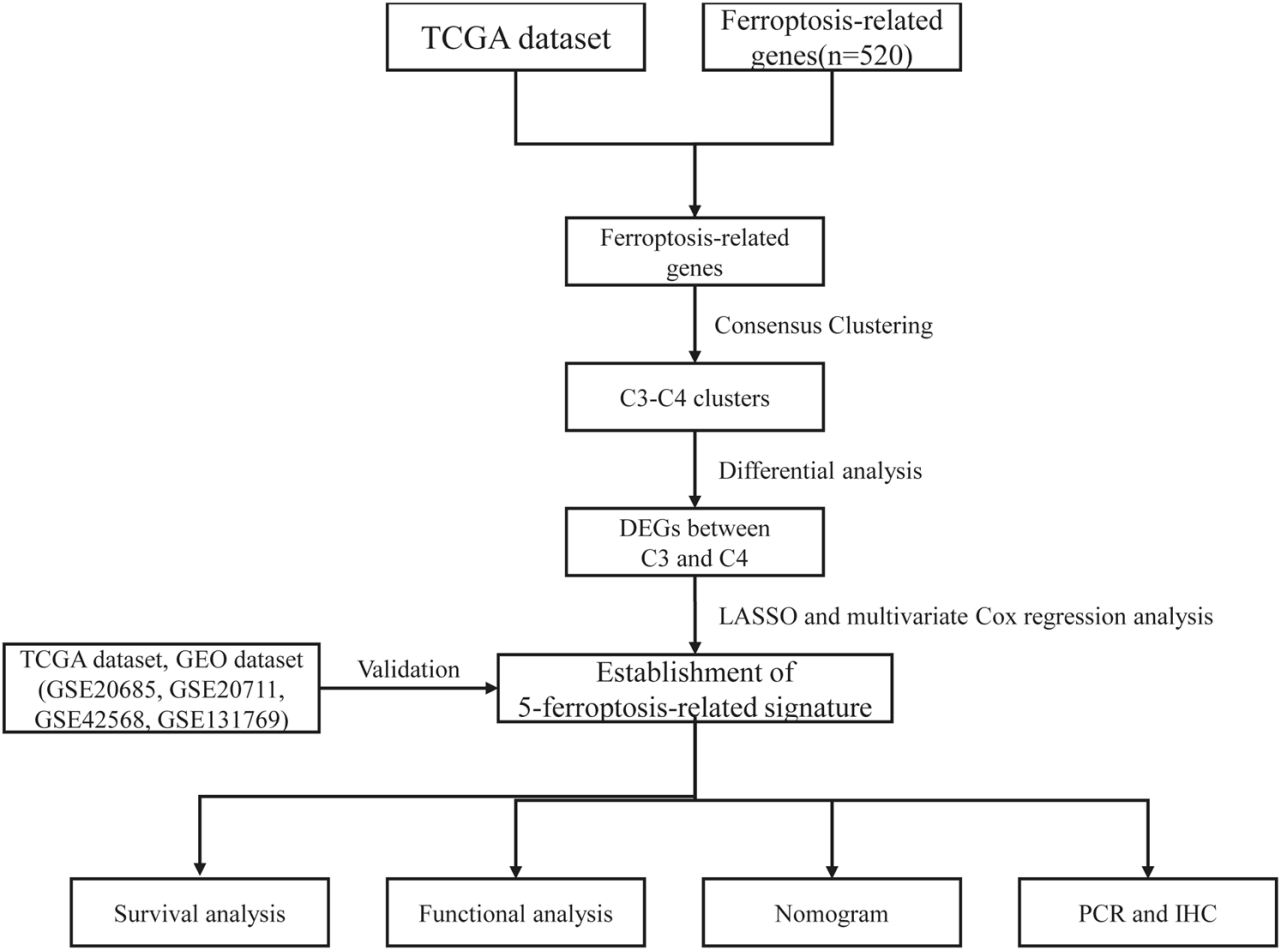
Overview of the process of this study

**Fig. 2:**
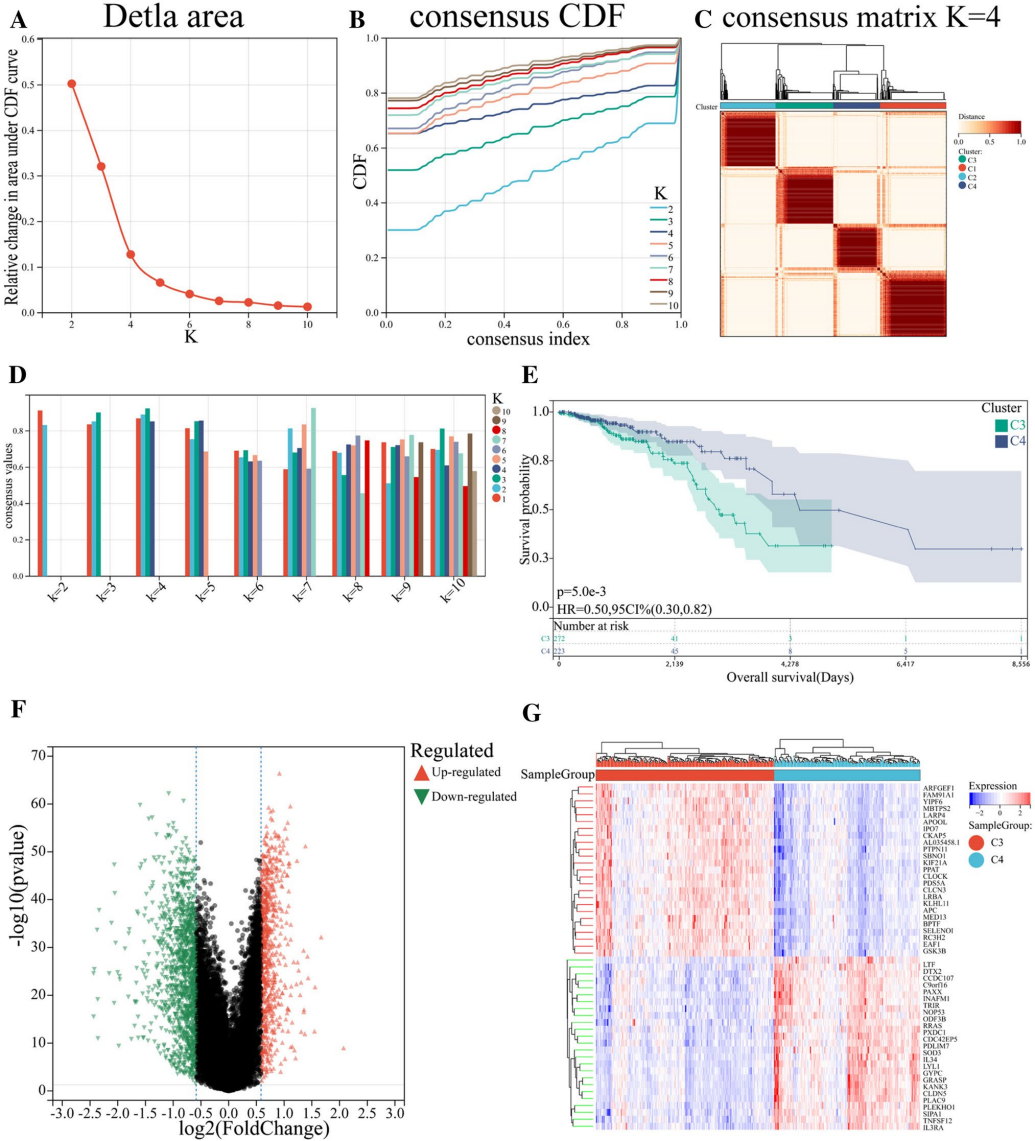
Clustering and identification of ferroptosis-related genes. **A** Relative change in area under CDF curve. **B** CDF curves of Ks (from 2 to 10). **C** Heat map showing sample clustering results, with consensus K identified as 4. **D** Clustering consistency plots for Ks (from 2 to 10). **E** Kaplan–Meier curves for cluster C3 and C4. **F** Volcano map of the differentially expressed ferroptosis-related genes between C3 and C4. Significantly upregulated and downregulated genes are shown in red and green, respectively. **G** Heat map of the differentially expressed ferroptosis-related genes between C3 and C4

Information of 1098 BCa samples and 113 normal breast tissues were downloaded from the TCGA database and compared with 520 ferroptosis-related genes from previous publications (Fig. 1). Based on the expression data of ferroptosis-related genes, CDF was applied to categorize the optimal number of clusters. When K was identified as 4, clustering results were relatively stable and CDF delta exhibited the slowest decreasing trend (Fig. 2A–B), indicating that the differences were most pronounced when BCa samples were clustered into four groups. In the present work, samples were divided into four groups: C1, C2, C3, and C4, as shown in the heat map (Fig. 2C). Besides, clustering consistency plots with other Ks (from 2 to 10) samples showed lower average intra-group consistency, with respect to the optimal number of clusters (Fig. 2D). To explore the survival difference between four clusters, Kaplan–Meier curves were performed. Although survival analysis of four groups revealed no significant difference (Supplementary Fig. 1), subgroup C3 had remarkable poorer prognosis than subgroup C4 (Fig. 2E), suggesting that survival difference could be attributed to the differentially expressed ferroptosis-related genes between C3 and C4. Volcano map and heat map of the differentially expressed ferroptosis-related genes between C3 and C4 were, therefore, plotted using the limma package in R language (Fig. 2F–G, Supplementary Table 2).

### Construction of ferroptosis-related model

**Fig. 3:**
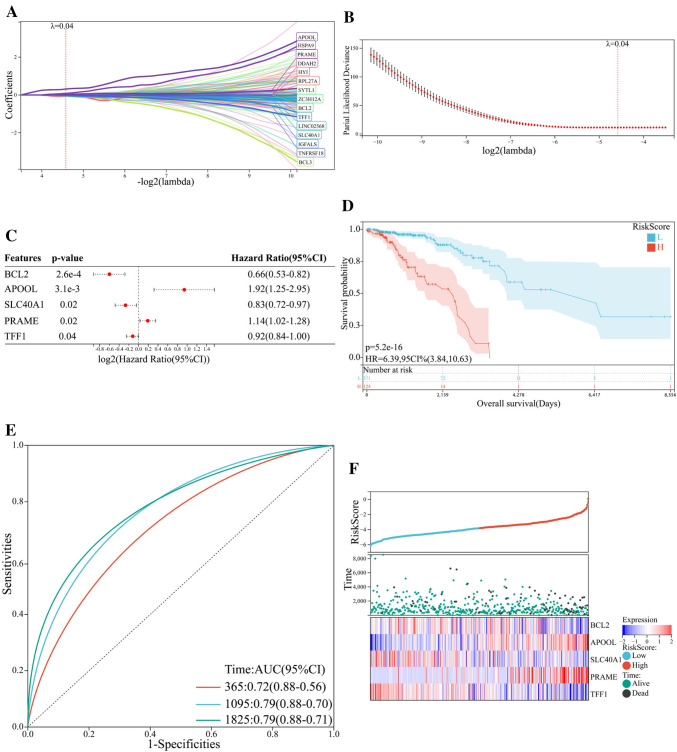
Construction of ferroptosis-related model. **A** LASSO regression analysis of 15 ferroptosis-related genes associated with prognosis. **B** Optimal penalty parameter λ identified by tenfold cross-validation. **C** Forest plot showing Cox regression analysis of five ferroptosis-related genes and overall survival. **D** Kaplan–Meier curves showing the overall survival of patients in high-risk group and low-risk group. **E** ROC curves of the five-gene signature prediction model. **F** Correlations among risk score, heat map of gene expression, and survival status of patients

Based on the preliminarily screened gene dataset (Supplementary Table 2), a prognostic risk evaluation model was constructed via LASSO analysis using the glmnet package in R language. After choosing the optimal penalty parameter, λ correlated with the minimum tenfold cross-validation, fifteen ferroptosis-related genes were identified as candidate genes (Fig. 3A–B). Prognostic significance of enrolled genes was then evaluated using the survival package in R language. Among 15 candidate ferroptosis-related genes, BCL2, SLC40A1, and TFF1 were associated with better prognosis; whereas APOOL and PRAME were related to poor prognosis (Fig. 3C). Based on the LASSO analysis and multivariate Cox regression analysis, these five ferroptosis-related genes were selected to construct the prognostic model. Risk score for each patient was obtained, and patients were stratified into high-risk group or low-risk group according to the optimal cutoff value − 3.137 calculated using the maxstat package in R language. Kaplan–Meier curve indicated that patients in high-risk group had a significantly worse overall survival, with respect to patients in low-risk group (Fig. 3D). Using the pROC package in R language, time‐dependent ROC analysis was performed to further evaluate the prediction efficiency of the constructed five-gene signature model, with the areas under curve (AUC) of 365, 1095, and 1825 days being 0.72, 0.79, and 0.79, respectively (Fig. 3E). Correlations among risk score, gene expression, and survival status were also analyzed. Patients in high-risk group expressed increased levels of APOOL and PRAME, and patients in low-risk group displayed elevated levels of BCL2, SLC40A1, and TFF1, suggesting that APOOL and PRAME were risk factors and the rest three ferroptosis-related genes were protective factors (Fig. 3F).

### Validation of the five-gene signature in TCGA and GEO dataset

**Fig. 4:**
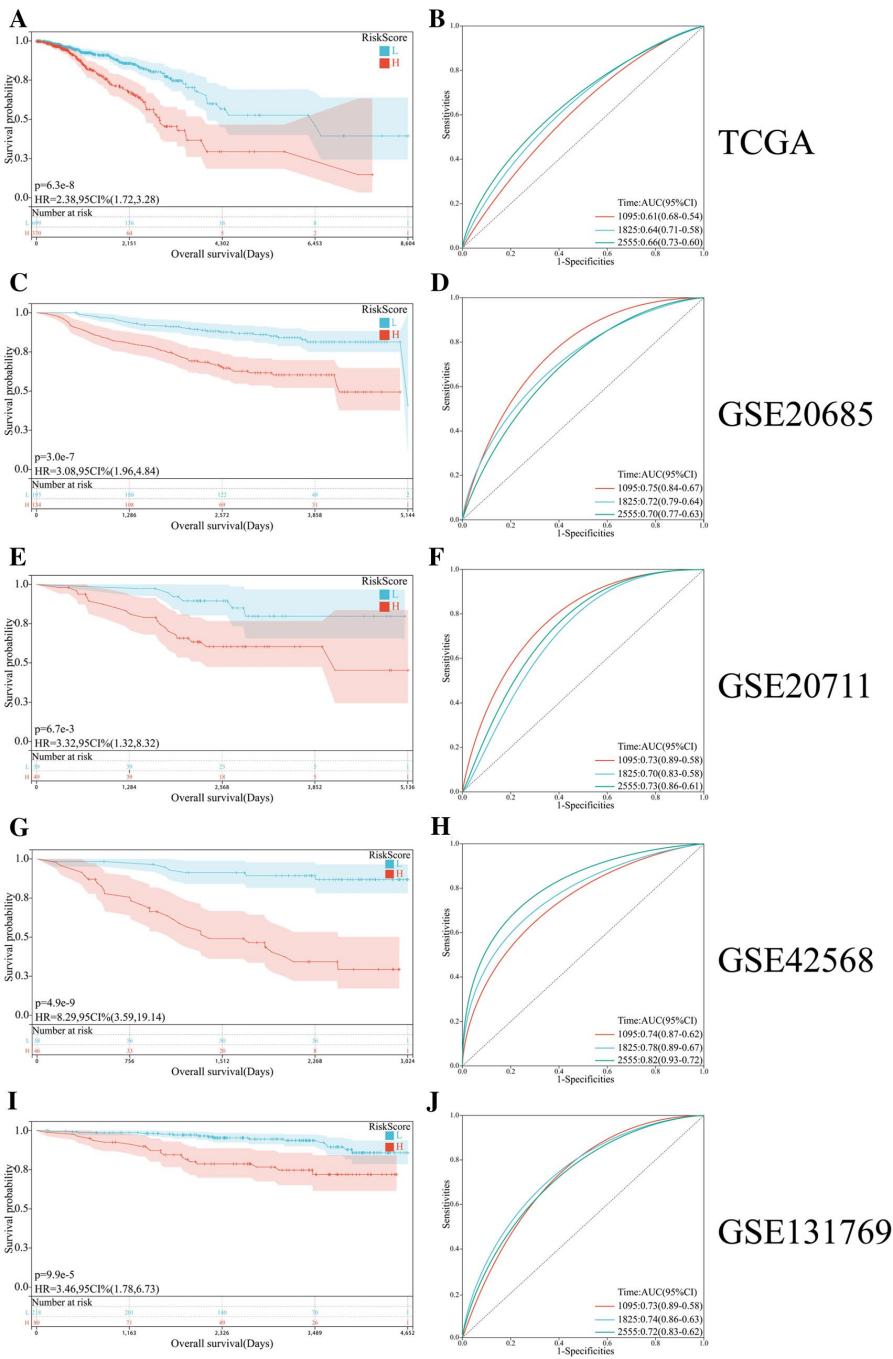
Validation of the five-gene signature model in the TCGA and GEO. Kaplan–Meier curves showing the overall survival of patients in high-risk group and low-risk group in TCGA (**A**), GSE20685 (**C**), GSE20711 (**E**), GSE42568 (**G**), and GSE131769 (**I**) cohorts. ROC curves of the five-gene signature model in TCGA (**B**), GSE20685 (**D**), GSE20711 (**F**), GSE42568 (**H**), and GSE131769 (**J**) cohorts

To test the robustness of the five-gene signature prediction model, patients from the TCGA, GSE20685, GSE20711, GSE42568, and GSE131769 cohorts were also categorized into high-risk or low-risk group by the optimal cutoff value calculated with the same formula. Consistent with the results described above, patients in high-risk group had a reduced survival time compared with those in low-risk group (Fig. 4A), and AUCs of the five-gene signature were 0.61 at 1095 days, 0.64 at 1825 days, and 0.66 at 2555 days (Fig. 4B) in the TCGA cohort. Likewise, patients in high-risk group had a poor prognosis, with respect to patients in low-risk group in GSE20685, GSE20711, GSE42568, and GSE131769 cohorts (Fig. 4C, E, G, and I). The AUCs for 1095 days, 1825 days, and 2555 days survival prediction were 0.75, 0.72, and 0.70 in the GSE20685 dataset (Fig. 4D), 0.73, 0.70, and 0.73 in the GSE20711 dataset (Fig. 4F), 0.74, 0.78, and 0.82 in the GSE42568 dataset (Fig. 4H), and 0.73, 0.74, and 0.72 in the GSE131769 dataset (Fig. 4J). These data indicated a good performance of the five-gene signature for survival prediction in BCa.

### Elucidation of molecular functions and pathways in the TCGA cohort

**Fig. 5:**
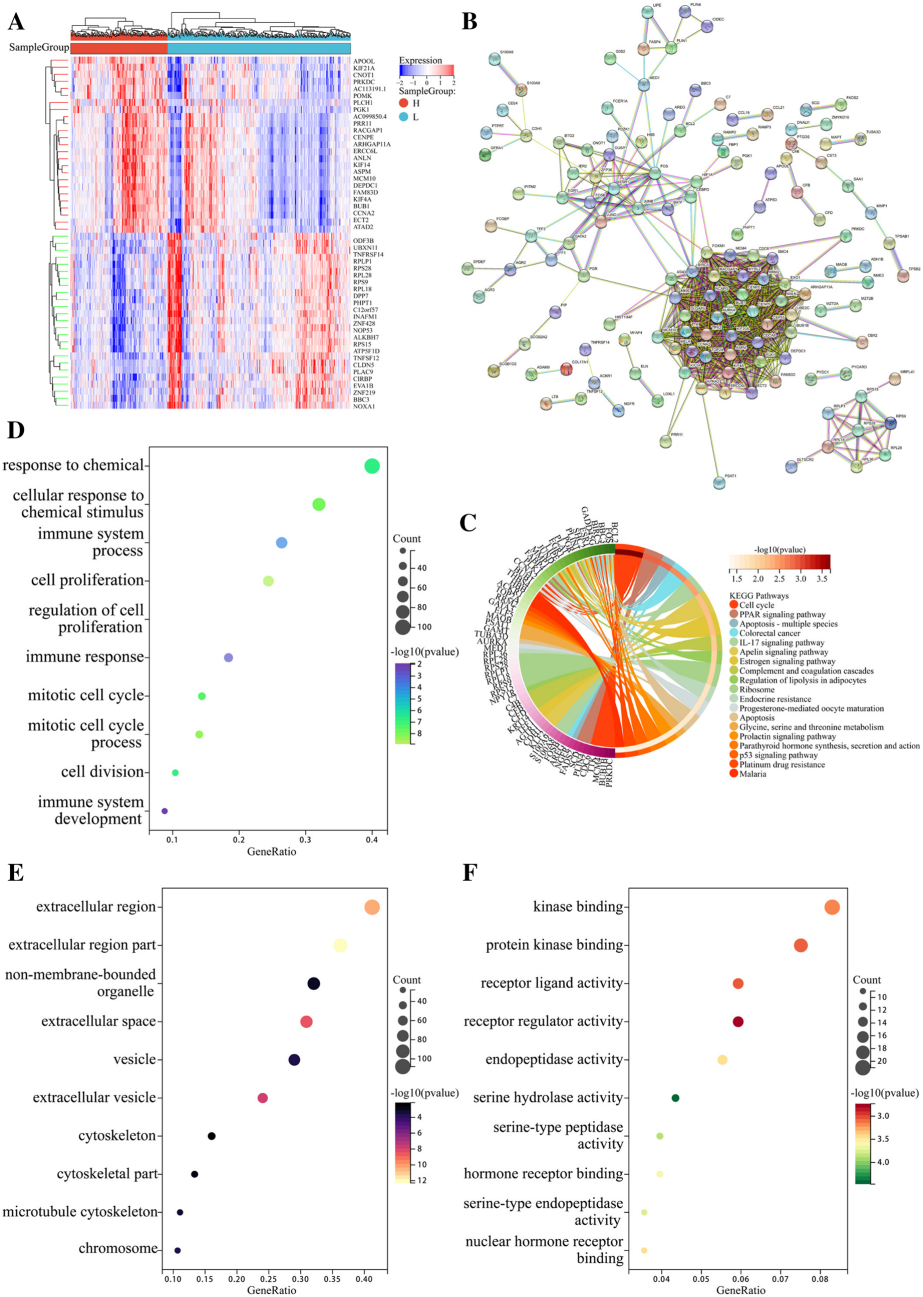
Elucidation of molecular functions and pathways. **A** Heat map of the differentially expressed genes between high-risk and low-risk groups. **B** Protein–protein interaction network of the differentially expressed genes between high-risk and low-risk groups downloaded from the STRING database. **C** KEGG analysis showing many cancer-related molecular pathways and drug resistance pathways. **D** GO analysis showing enrichment of biological processes. **E** GO analysis showing enrichment of cell components. **F** GO analysis showing enrichment of molecular functions

To explore the underlying difference in molecular functions and signaling pathways between high-risk and low-risk groups classified by the five-gene signature, gene set enrichment analysis was performed. First, patients were divided into high-risk group or low-risk group based on the optimal cutoff value 1.307 calculated using the maxstat package in R language. Heat map of the differentially expressed genes between high-risk and low-risk groups was then plotted using the limma package in R language (Fig. 5A, Supplementary Table 3). An interaction network of differentially expressed genes between the two groups was generated using STRING (Fig. 5B). KEGG analysis showed that many cancer-related molecular pathways were enriched, including cell cycle in cancer, p53 signaling pathway, and estrogen signaling pathway. PPAR signaling pathway and IL-17 signaling pathway were associated with immune response. Pathways related to drug resistance, such as endocrine resistance and platinum resistance, were also involved (Fig. 5C). GO analysis was conducted using the clusterProfiler package in R language and indicated the enrichment of biological processes (Fig. 5D), cell components (Fig. 5E), and molecular functions (Fig. 5F). Interestingly, several immune-related biological processes were significantly enriched, including immune system process, immune response, and immune system development. These results suggested that risk score of the five-gene signature was mainly related to cancer progression, drug resistance, and tumor immunity.

### Association between gene signature and clinicopathological features

**Fig. 6:**
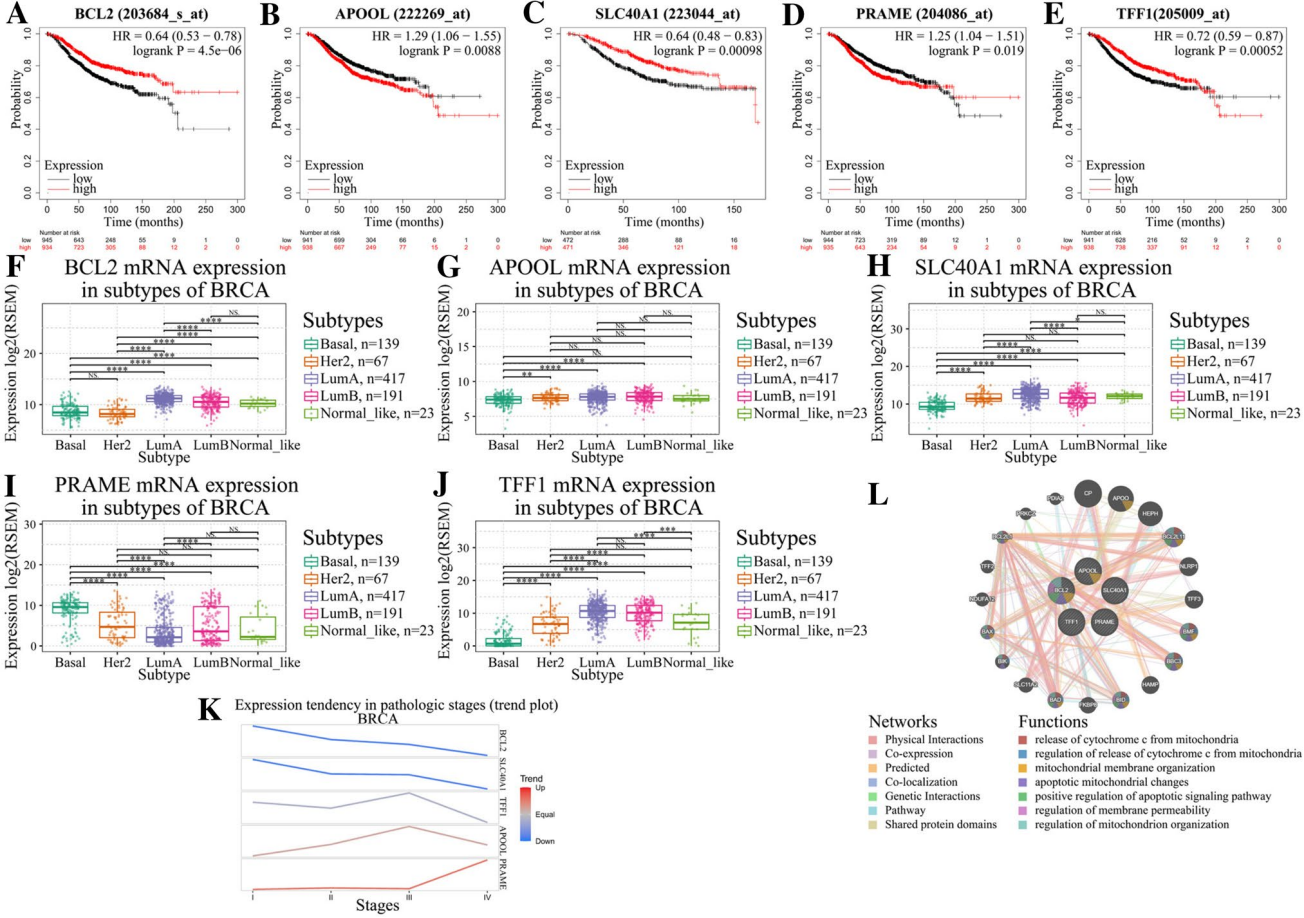
Association between signature and clinicopathological features. Kaplan–Meier curves showing the overall survival of patients expressed high or low level of BCL2 (**A**), APOOL (**B**), SLC40A1 (**C**), PRAME (**D**), and TFF1 (**E**). Relationship between different subtypes of BCa and expression levels of BCL2 (**F**), APOOL (**G**), SLC40A1 (**H**), PRAME (**I**), and TFF1 (**J**). **K** Trend plot showing gene expression tendency in different pathological stages of BCa. **L** Prediction and analysis of genes that were functionally similar to these five genes

Correlations between the five-gene signature and clinical features were analyzed. Kaplan–Meier plotter demonstrated that higher expression of BCL2, SLC40A1, and TFF1 was significantly associated with longer overall survival in BCa patients, whereas expression levels of APOOL and PRAME were negatively related to prognosis (Fig. 6A–E). Relationship between the five-gene signature and different subtypes of BCa was obtained through the GSCA database. Either protective factors BCL2, SLC40A1, and TFF1 or risk factors APOOL and PRAME were differentially expressed in different subtypes of BCa (Fig. 6F–J). Interestingly, significant difference of BCL2 expression level was found among Her2, luminal A, and luminal B subtypes of BCa, but no significant difference of APOOL gene expression was observed among Her2, luminal A, and luminal B subtypes of BCa. PRAME was highly expressed in basal-like BCa, whereas TFF1 was relatively increased in luminal A subtype. In addition, BCL2, SLC40A1, and TFF1 showed a decreasing expression trend with the increase of BCa pathological stage, and APOOL and PRAME showed an increasing trend (Fig. 6K). This, along with the above survival curves, indicated that the five ferroptosis-related genes were significantly correlated with clinicopathological parameters. GeneMANIA database was used to predict the genes that were functionally similar to these five genes. A total of 20 genes were obtained, and function analysis showed that they were mainly involved in mitochondria biological functions (Fig. 6L). As a matter of fact, reduction or disappearance of mitochondrial cristae, mitochondrial outer membrane rupture, and mitochondrial membrane condensation were all morphological changes of ferroptosis.

### Functional study of the five-gene signature

**Fig. 7:**
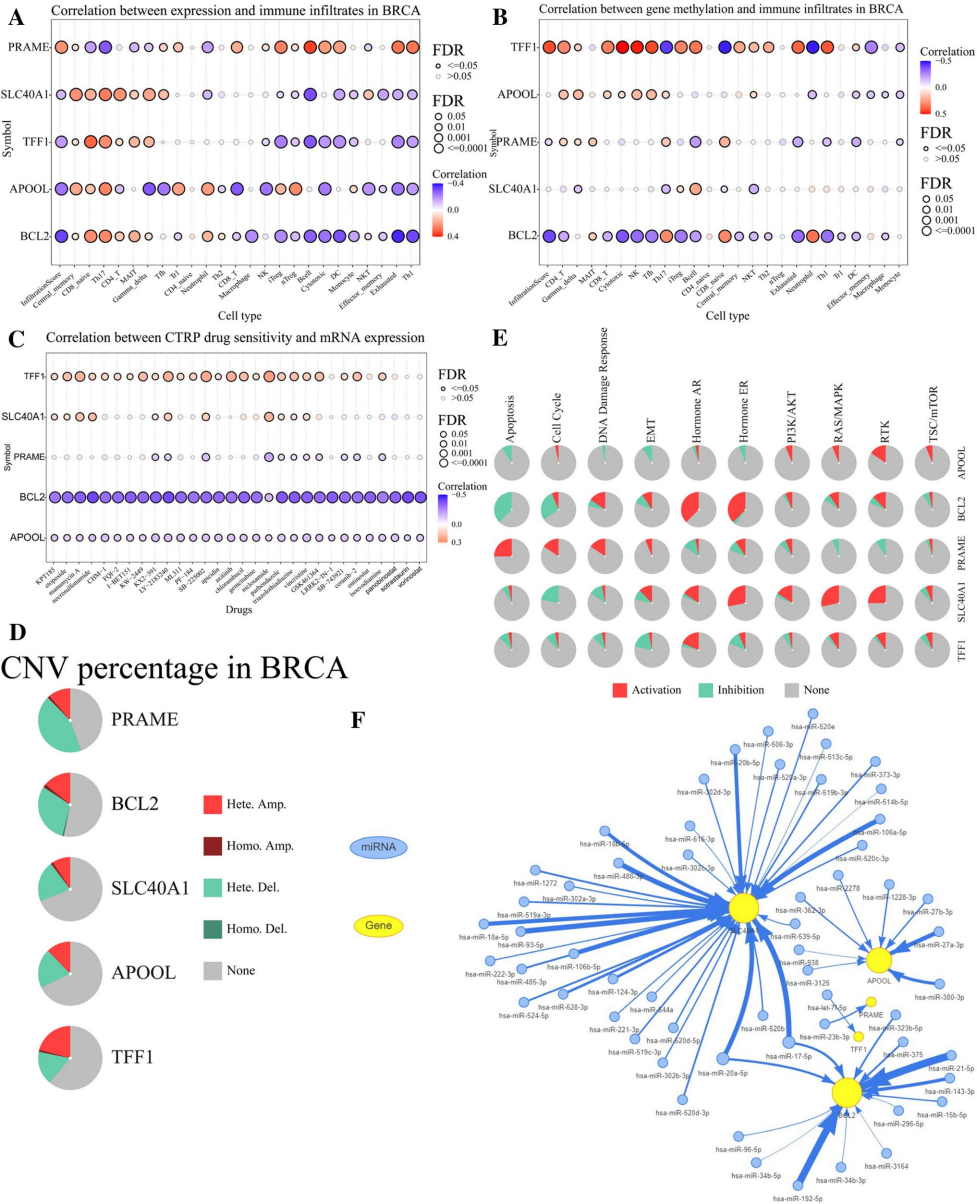
Functional study of the five signature genes. **A** Correlation between gene expression and immune infiltration in BCa. **B** Correlation between gene methylation and immune infiltration in BCa. **C** Correlation between gene expression and drug sensitivity (top 30) in pan-cancer. **D** Pie plot showing the copy number variation percentage of the five signature genes in BCa. Hete. (heterozygous); Homo. (homozygous); Amp. (amplification); Dele. (deletion). **E** Pathway activated or inhibited by the five signature genes. **F** Upstream miRNA regulatory network

Both the GSCA and GSCALite databases were applied to analyze the biological functions of the five-gene signature. Regarding immune infiltration, BCL2, APOOL, SLC40A1, and TFF1 were negatively correlated with the infiltration of Th1, Exhausted T cells, dendritic cells, Cytotoxic T cells, and B cells, but were positively correlated with the infiltration of Central_memory T cells, CD8_naive T cells, and Th17. However, PRAME was positively correlated with the infiltration of Th1, Exhausted T cells, dendritic cells, Cytotoxic T cells, and B cells, and was negatively correlated with the infiltration of CD8_naive T cells, and Th17 (Fig. 7A). Since methylation alters gene expressions and functions, significance of gene methylation in immune infiltration was investigated. Specifically, BCL2 and PRAME methylation were negatively correlated with immune infiltration, whereas TFF1 was positively correlated with immune infiltration (Fig. 7B). In terms of the five-gene signature and drug sensitivity, BCL2 and APOOL were negatively correlated with the sensitivity of chemotherapy drugs commonly used in BCa, such as etoposide, gemcitabine, and vincristine. Conversely, TFF1 was positively correlated with chemosensitivity (Fig. 7C). In addition, BCL2 and PRAME showed the highest copy number variation percentage, with total amplification percentage and total deletion percentage of 15.74% and 31.11% for BCL2, 12.41% and 42.96% for PRAME (Fig. 7D and Supplementary Table 4). Pathway activity map showed that BCL2 strongly activated androgen receptor and estrogen receptor but inhibited apoptotic pathway, whereas PRAME activated apoptotic pathways. SLC40A1 activated estrogen receptor, RAS/MAPK, and RTK pathway but inhibited cell cycle (Fig. 7E). To obtain the potential upstream miRNAs of the five ferroptosis-related genes, upstream miRNA regulatory network containing miR-21-5p, miR-192-5p, miR-143-3p, miR-488-3p, miR-106a-5p, miR-20a-5p miR-17-5p, miR-20b-5p, miR-18a-5p, miR-93-5p, miR-18b-5p, miR-27a-3p, miR-380-3p was mapped (Fig. 7F).

### Expression of the five ferroptosis-related genes at gene and histological level

**Fig. 8:**
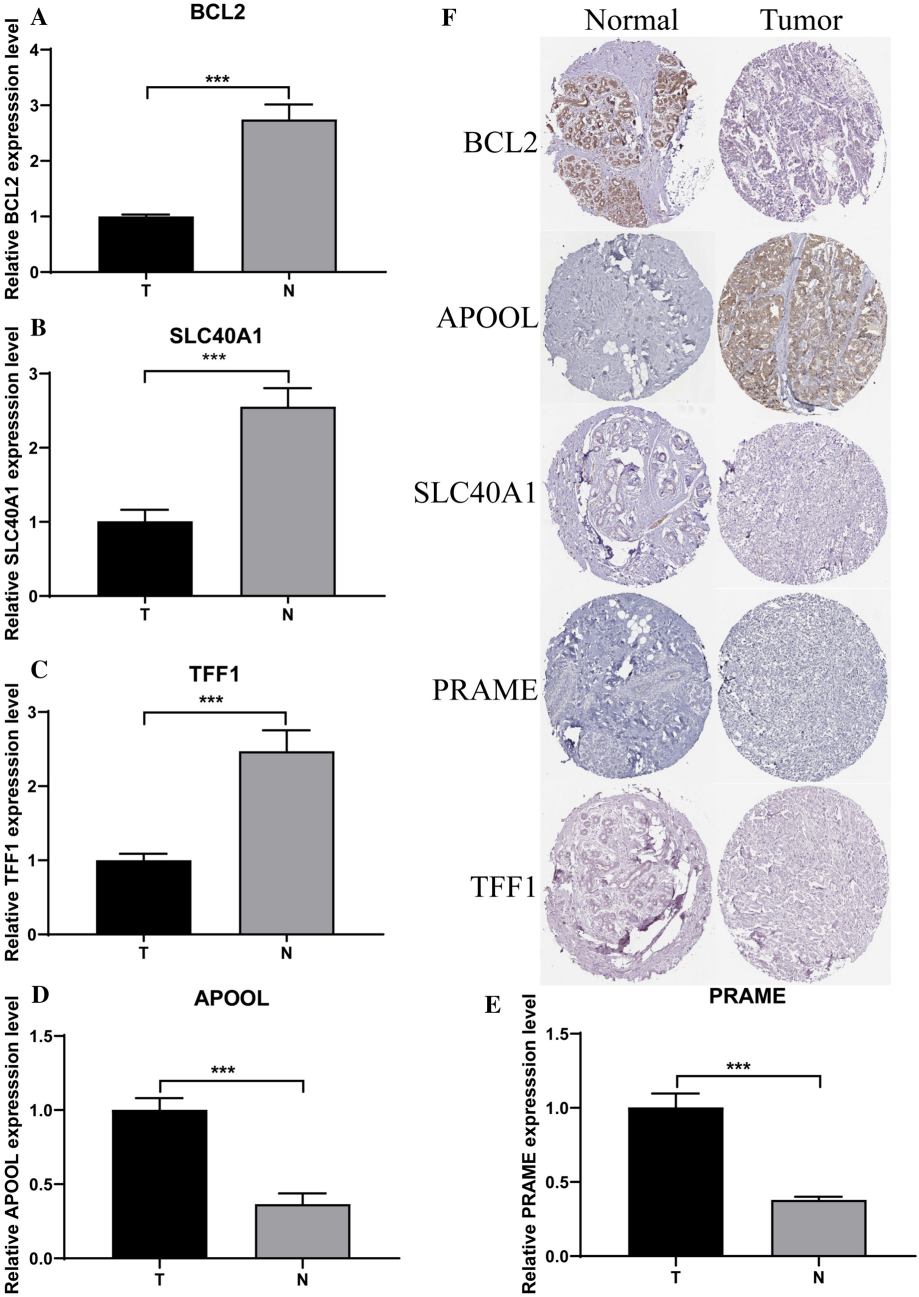
Expression of the five ferroptosis-related genes at gene and histological level. Expression levels of BCL2 (**A**), SLC40A1 (**B**), TFF1 (**C**), APOOL (**D**), and PRAME (**E**) in five pairs of BCa samples and adjacent normal breast tissues obtained from own hospital. *** *P* < 0.001. **F** Immunohistochemical analysis in the HPA database showing the expressions of BCL2, APOOL, SLC40A1, PRAME, and TFF1 proteins in BCa tissues and normal tissues

Gene expressions of the five ferroptosis-related genes in BCa samples and adjacent normal tissues were analyzed. Specifically, expression levels of BCL2, SLC40A1, and TFF1 were decreased in BCa samples with respect to normal tissues, whereas APOOL and PRAME were increased in tumor samples (Fig. 8A–E). According to the immunohistochemical analysis in the HPA database (Fig. 8F), the low staining intensity of BCL2 in tumor tissues contrasted sharply with the high staining intensity in normal tissues, whereas the high staining intensity of APOOL in tumor tissues contrasted sharply with the low staining intensity in normal tissues. SLC40A1 showed low staining intensity in normal tissues but lacked staining in tumor tissues. However, PRAME and TFF1 did not show significant differences at histological level.

### Construction of a predictive nomogram

**Fig. 9:**
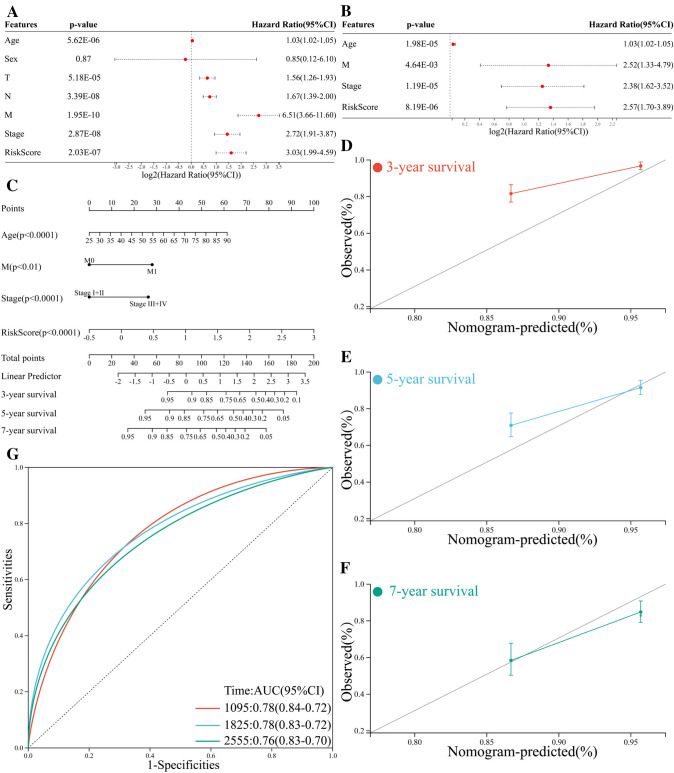
Construction of a predictive nomogram for BCa patients based on risk score and clinical features. **A** Forrest plot of the univariate Cox regression analysis. **B** Forrest plot of the multivariate Cox regression analysis. **C** Nomogram for predicting 3-year, 5-year, and 7-year overall survival of BCa patients. Calibration curves for predicting 3-year (**D**), 5-year (**E**), and 7-year (**F**) overall survival of BCa patients. **G** ROC curves for 3-year, 5-year, and 7-year overall survival of the nomogram

**Fig. 10:**
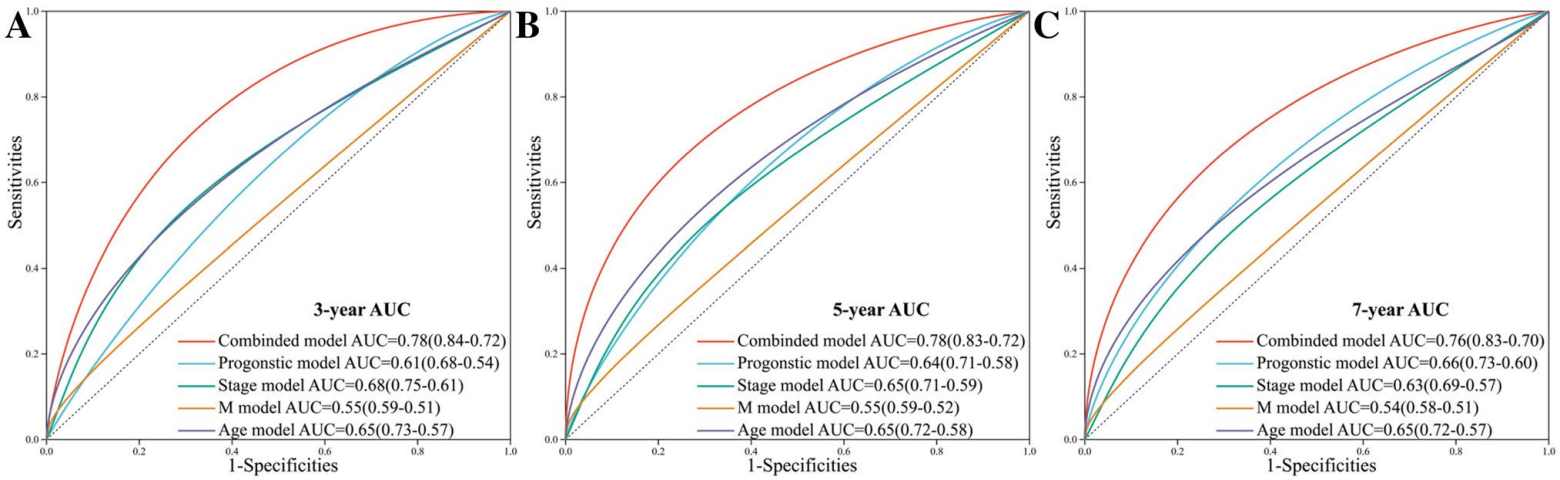
Comparison of the nomogram. Nomogram with age, metastasis status, clinical stage, the five-gene prognostic signature, and the combined model for predicting 3-year (**A**), 5-year (**B**), and 7-year (**C**) overall survival of BCa patients

Univariate and multivariate analysis revealed significant correlations between overall survival of BCa patients, and risk score and several clinical features including patient age, distant metastasis, and tumor stage (Fig. 9A–B). To build a predictive method, a prognostic nomogram was generated to predict 3-year, 5-year, and 7-year overall survival of BCa using the RMS package in R language (Fig. 9C). Calibration curves showed the prediction value of the nomogram and demonstrated high accuracy of the predicted survival (Fig. 9D–F). The AUCs of nomogram were 0.78, 0.78, and 0.76 for 3-year, 5-year, and 7-year, respectively (Fig. 9G). Compared with nomogram including only age, metastasis status, clinical stage, or the five-gene prognostic signature, the combined model showed the largest AUCs for 3-year, 5-year, and 7-year overall survival (Fig. 10A–C). Taken together, these results indicated that compared with nomogram built with a single prognostic factor, the nomogram established with the combined model might be a promising method for predicting overall survival for BCa patients.

## Discussion

BCa remains a major challenge for women's health worldwide (Giaquinto et al. 2022). Clinical, pathological, and molecular features help predict treatment response and clinical outcomes in some degree. However, given that BCa is a heterogeneous group of diverse subtypes with own biologic characteristics, discovery of novel prognostic biomarkers and establishment of more accurate prognostic models are of crucial importance in BCa research (Duffy et al. 2015). Recently, signatures based on the differentially expressed ferroptosis-related genes have gained much attention and shown great potential in prognosis prediction of cancer (Liang et al. 2020; Liu et al. 2019; Wu et al. 2021).

In the present study, a novel five-gene signature including BCL2, SLC40A1, TFF1, APOOL, and PRAME was identified from a number of ferroptosis-related genes and then established for prognosis prediction in BCa. While BCL2, SLC40A1, and TFF1 were positive prognostic genes, APOOL and PRAME were found to do the opposite. Risk score based on the five-gene signature was an independent prognostic factor of BCa and patients in high-risk group had a significantly worse overall survival, with respect to patients in low-risk group. According to the survival curves and AUCs values, prediction performance of the five-gene signature was good not only in the TCGA cohort but also in the GSE20685, GSE20711, GSE42568, and GSE131769 cohorts. Additionally, ROC demonstrated that the nomogram combining the five-gene signature with clinical features including patient age, distant metastasis, and tumor stage performed the best in predicting 3-year, 5-year, and 7-year overall survival for BCa patients. All these results indicated that the prediction model developed from the five ferroptosis-related genes could be a promising indicator for BCa survival.

Cancer progression mainly depends on the balanced levels of pro-apoptotic proteins, such as Bax, and anti-apoptotic proteins, including BCL2. Numerous studies have shown that BCa patients with increased BCL2 level are more responsive to endocrine therapy and have more favorable overall survival (Zhang et al. 1998). Disruption of BCL2 expression could cause apoptosis and induce ferroptosis in lymphoma (Setiawan et al. 2023). Besides, traditional Chinese medicine was found to inhibit the viability of lung cancer cells and induce ferroptosis by promoting Bax via inhibiting BCL2 (Huang et al. 2022). SLC40A1, the only discovered mammalian iron efflux transporter in the plasma membrane, mediates intracellular iron exportation. SLC40A1 deficiency was related to intracellular iron accumulation, and several studies demonstrated that inhibiting SLC40A1 significantly prevented tumor cell growth by inducing ferroptosis (Xu et al. 2022; Tang et al. 2021). Moreover, ferroptosis suppressor SLC40A1 was significantly associated with prognosis and immunosuppression, serving as a survival predictor in glioblastoma and pancreatic cancer (Deng et al. 2021; Feng et al. 2021). TFF1, a small secretory protein frequently expressed in BCa, has been associated with bone metastasis and even been regarded as an oncogene (Smid et al. 2006). However, controversial data have also suggested a beneficial role of TFF1 in BCa. For example, Yi et al. demonstrated that TFF1 was able to inhibit proliferation, migration, and invasion of BCa cells in vitro (Yi et al. 2020). Buache et al. confirmed that TFF1 did not exhibit oncogenic properties, but rather reduced BCa development in vivo (Buache et al. 2011). Corte et al. reported that TFF1 level was related to a better clinical outcome, especially in those with node-negative BCa (Corte et al. 2006). PRAME, a cancer testis antigen first isolated in tumor-reactive T cell clones from metastatic melanoma, is highly abundant and acts as a prognostic marker for several cancers including melanoma and ovarian adenocarcinoma (Haqq et al. 2005; Partheen et al. 2008). In BCa, PRAME could increase cancer cell motility through reprogramming genes of epithelial-to-mesenchymal transition, thus promoting BCa progression (Al-Khadairi et al. 2019). Additionally, PRAME expression was significantly associated with enhanced distant metastasis and reduced overall survival, serving as a useful prognostic and predictive marker for BCa (Doolan et al. 2008). APOOL is a cardiolipin-binding constituent of the Mitofilin/MINOS protein complex. Weber et al. confirmed that over-expression of APOOL resulted in mitochondria fragmentation, decreased basal oxygen consumption rate, and altered cristae morphology. Conversely, downregulation of APOOL impaired mitochondrial respiration and led to cristae morphology alterations (Weber et al. 2013). APOOL was recently reported to be a prognostic and therapeutic marker in esophageal carcinoma and glioblastoma (Zhang et al. 2023; Serão et al. 2011). Since few related studies on these genes except for BCL2 and SLC40A1 have been reported, whether the above mentioned five genes play a vital role in overall survival of BCa patients through promoting or preventing ferroptosis remains to be fully elucidated.

Although accumulating evidence has revealed that ferroptosis is closely related to tumor immune microenvironment, the potential relationship between ferroptosis and tumor immunity remains unclear. In the present study, GO enrichment and KEGG pathway analysis were performed by analyzing the differentially expressed genes between high-risk and low-risk groups classified by the five-gene signature. Particularly, PPAR signaling pathway and IL-17 signaling pathway were discovered in KEGG analysis. PPAR signaling pathway was involved in the innate and adaptive immune system and has been implicated in immune response (Christofides et al. 2021). Enhanced IL-17 signal transduction was associated with the increase of CD8^+^ T cell infiltration and variation of the BCa immune microenvironment (Shuai et al. 2020). In addition, several immune-related biological processes were significantly identified in GO enrichment. These gene enrichment analyses collectively suggested that ferroptosis might be closely related to immune response of BCa.

To the best of knowledge, the five ferroptosis-related gene signature and nomogram have not been reported previously and could be a promising prognostic model for BCa. In particular, APOOL, PRAME, and TFF1 were first applied in gene signature for prognosis prediction for BCa. Nomogram combining the present five-gene signature with several conventional clinical features including patient age, distant metastasis, and tumor stage showed significantly improved performance in predicting overall survival. The AUCs of nomogram were 0.78, 0.78, and 0.76 for 3-year, 5-year, and 7-year, respectively, and were relatively higher than some other published nomogram for BCa prediction (Wu et al. 2021; Wang et al. 2022). Several previous studies also used similar methods and did not validate the nomogram (Wang et al. 2021; Zhu et al. 2021), indicating the feasibility and validity of the present work. However, several limitations of the present work should be taken into consideration. First, the five ferroptosis-related gene signature and prognostic model were established with retrospective data from public databases. More prospective multicenter data are warranted for validation in the future. Second, the specific functions and mechanisms of the five ferroptosis-related genes in BCa remain elusive, and more thorough researches are now needed to clarify the significance and mechanism of the five genes. Third, this model only analyzed overall survival as an indicator of prognosis, disease-free survival, and short-term survival could be included as well. Four, except its excellent performance in predicting BCa survival, the performance of the five-gene signature in predicting survival of different BCa subtypes remains to be deeply elucidated. Five, there may be many factors that affect patient survival such as gene methylation, copy number variations, and single nucleotide polymorphisms; it is, therefore, desired that further attention be drawn to this field.

## Conclusion

In summary, the present study established a novel five-gene signature and nomogram to predict overall survival of BCa, which may not only be used for clinical management of patients but also accelerate future studies investigating ferroptosis as the therapeutic target in BCa.

## Supplementary Information

Below is the link to the electronic supplementary material.

Supplementary file1 (TIF 18440 KB) Supplementary Figure 1. Kaplan-Meier curves for different groups. Kaplan-Meier curves showing overall survival of four groups (A), cluster C1 and C2 (B), cluster C1 and C3 (C), cluster C1 and C4 (D), cluster C2 and C3 (E), and cluster C2 and C4 (F)

Supplementary file2 (XLSX 14 KB)

Supplementary file3 (XLSX 223 KB)

Supplementary file4 (XLSX 41 KB)

Supplementary file5 (XLSX 9 KB)

## Data Availability

The datasets analyzed during the current study are available from the corresponding author on reasonable request.
