# Supplementary figures and images for: Identification of a novel five ferroptosis-related gene signature as a promising prognostic model for breast cancer
Source: J Cancer Res Clin Oncol. 2023 Sep 20;149(18):16779–95. doi: 10.1007/s00432-023-05423-5 (PMC10645672; doi:10.1007/s00432-023-05423-5)

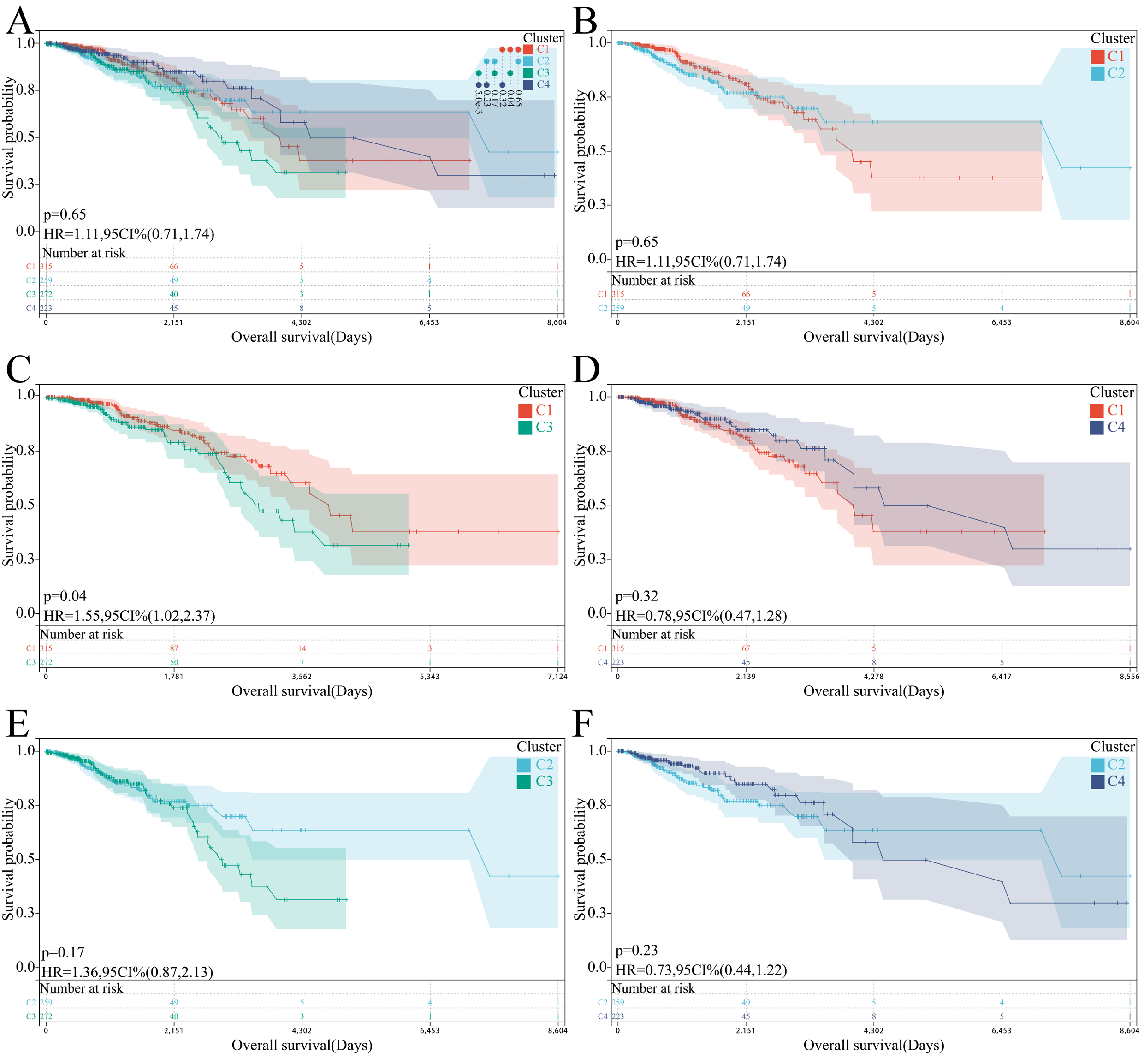

Supplement: Supplementary file 1 — Supplementary file1 (TIF 18440 KB) Supplementary Figure 1. Kaplan-Meier curves for different groups. Kaplan-Meier curves showing overall survival of four groups (A), cluster C1 and C2 (B), cluster C1 and C3 (C), cluster C1 and C4 (D), cluster C2 and C3 (E), and cluster C2 and C4 (F) [file 432_2023_5423_MOESM1_ESM.tif]
